# Prevalence of and Factors Associated With Eustachian Tube Dysfunction Among the Public in Taif, Saudi Arabia

**DOI:** 10.7759/cureus.27482

**Published:** 2022-07-30

**Authors:** Wahaj A Altalhi, Abeer I Alsulaimani, Zouhor A Alhossaini, Rawan M Alharthi, Zohour A Almalki, Wjood A Altalhi, Shrooq H Alswat, Ghaliah O Alnefaie

**Affiliations:** 1 Medicine, Taif University, Taif, SAU; 2 Family Medicine, Ministry of Health, Taif, SAU; 3 General Practice, Ministry of Health, Taif, SAU

**Keywords:** aural fullness, tinnitus, ear pressure, otalgia, ear pain, eustachian tube dysfunction

## Abstract

Background

The eustachian tube is a tubular structure connecting the middle ear cavity with the nasopharynx, providing ventilation and pressure equalization in the middle ear, mucociliary clearance, and preventing the reflux of sound and fluid from the nasopharynx. Therefore, any kind of deficiency in this tube will lead to eustachian tube dysfunction (ETD).

Aim

This study aims to evaluate the prevalence and associated factors of ETD in the Taif public.

Materials and methods

A descriptive cross-sectional survey was conducted among the public in Taif, Saudi Arabia and it was done during the period between September 7 and September 28, 2021. A predesigned online questionnaire in the Arabic language was used to collect the data. The questionnaire contained three main parts. The first one included demographic questions, the second risk factors, and the third manifestations of ETD. The questionnaire was initiated after a literature review of similar articles and after experts' consultation for validity and reliability.

Results

A total of 693 participants completed the study questionnaire. The exact 546 (78.8%) participants were females and 671 (96.8%) were Saudi. The exact 122 (61%) participants among those who had ETD or hearing loss reported improvement in their symptoms after moving to another city below sea level. The exact 146 (21.1%) participants had ETD while 547 (78.9%) had reported normal function. ETD was detected among 69.2% of those who were previously diagnosed with ETD versus 20.1% of others (P=0.001). Participants with a family history of hearing loss showed a nearly doubled risk for having ETD (OR=1.98; 95% CI: 1.33-2.94) and smokers had nearly the same doubled likelihood(OR=1.83; 95% CI: 1.01-3.48).

Conclusion

In conclusion, our study revealed a slightly high prevalence of ETD and hearing loss among the public in Taif, Saudi Arabia, and the factors associated with ETD.

## Introduction

The eustachian tube is a tubular structure that connects the middle ear cavity with the nasopharynx, providing ventilation and pressure equalization in the middle ear, mucociliary clearance, and prevention of the reflux of sound and fluid from the nasopharynx. Therefore, any kind of deficiency on this tube will lead to eustachian tube dysfunction (ETD) which is a common diagnosis in some conditions where the eustachian tube is unable of performing its functions adequately, resulting in negative pressure, muffled hearing “popping” sounds, tinnitus, aural fullness and otalgia [1,2].

The ETD and related conditions are associated with 2 million clinic visits per year among patients above 20 years of age and 2.6 million visits among those under 20 years of age [3]. A recent study was conducted in Jeddah, Saudi Arabia with 2,372 participants and showed the prevalence of ETD at approximately 42.49%, also mentioned the risk of developing ETD more in females than males [4].

Furthermore, ETD might lead to various middle ear disorders such as otitis media with effusion (OME), tympanic membrane atelectasis, and cholesteatoma. Also, it has long-term impacts, like communication difficulties and decreased productivity, which can contribute to low quality of life [2]. In adults, ETD can occur alone or in association with a variety of inflammatory illnesses of the aerodigestive tract [5].

Environmental factors and cigarette smoking has been suggested as possible cause of ETD, due to a disruption in the eustachian tube's mucociliary clearance mechanism. Preliminary studies into the risk factors for ETD in children and the need for tympanostomy tube placement for the treatment of RAOME found that children who lived in households where at least one parent smoked were more likely to require surgical management of OME [6]. Treatments of ETD range from advice and initial observation to be followed by pharmaceutical therapies such as steroids and in certain cases, a surgical referral [7].

A combination of clinical history, physical examination, tympanometry, audiometry, and other tests are used to determine ET dysfunction [8]. Although identifying acceptable diagnostic procedures and testing, as well as establishing criteria for identifying patients with ETD has always been challenging. Several tools for evaluating eustachian tube function have been reported. The use of eustachian tube function testing is limited; however, by the need for expensive equipment and qualified professionals, both of which are frequently available at specialist health care centers [4].

The seven-item Eustachian Tube Dysfunction Questionnaire (ETDQ-7) has recently been validated and hailed as having great discrimination against patients with ETD. It consists of seven questions, each graded on a Likert scale ranging from 1 (no problem) to 7 (severe problem). While the maximum total score is 49, it has been observed that a score of 14.5 provides 100% sensitivity and 100% specificity for ETD [9]. Knowledge of the prevalence of disease in the population is valuable in establishing possible community service needs now and in the future. To the best of our knowledge, studies in Saudi Arabia do not so far assess the prevalence of ETD in the community. This study aims to evaluate the prevalence and associated factors of ETD in the Taif public.

## Materials and methods

A cross-sectional study was done to assess the prevalence of and factors associated with ETD among the public in Taif, Saudi Arabia. Over the period from September 7 to September 28, 2021. A total sample size of 693 participants was involved from only the population living in Taif city, Saudi Arabia, who agreed to participate in the study, while participants other than the Taif city population, living outside Taif city and those who disagreed to participate were excluded.

Data collection instrument

We used a predesigned questionnaire. The questionnaire was designed in Arabic including, gender, age of the participant, City, whether the participant has been diagnosed with hearing loss or ETD, and family history of hearing loss. Also, it included eight questions about the symptoms of hearing loss by using ETDQ-7 was designed by McCoul et al. [10] as a new scoring system for the evaluation of the symptoms associated with obstructive ETD. Each of the seven items has scores ranging from a minimum of 1 to a maximum of 7 points, resulting in a total composite mean score of between 1 and 7 points by dividing the overall score by 7. A mean score≥2.5 is considered abnormal, with higher scores indicating a greater level of symptom severity [11]. The ETDQ-7 was used after validation and translation to Arabic and distributed among participants preceded by a brief explanation of the aim of the study. After the validation, the questionnaire was sent to the participants through various Social Media platforms (WhatsApp, Twitter).

Ethical considerations

Ethical approval was obtained from the research ethics committee of Taif University application no. 43-009.

Data analysis

After data were extracted, it was revised, coded, and entered into statistical software IBM SPSS version 22 (SPSS Inc., Chicago, IL). All statistical analysis was done using two-tailed tests. P-value less than 0.05 was statistically significant. Descriptive analysis based on the frequency and percent distribution was done for all variables including participants' demographic data, family history of hearing loss, medical diagnosis of ETD, improvement of ETD symptoms by changing high altitude residence, and smoking. Also, items on ETDQ-7 were shown in frequency tables. Crosstabulation was used to show ETD among participants by their socio-demographic data. Significance of relation was assessed using the Pearson chi-square test and exact probability test for small frequency distributions. A multiple logistic regression model was used to assess the most significant predictors of having ETD among study participants.

## Results

A total of 693 participants fulfilling the inclusion criteria completed the study questionnaire. Participants' ages ranged from 16 to more than 40 years with a mean age of 25.1 ± 12.9 years old. The exact 546 (78.8%) participants were females and 671 (96.8%) were Saudi. Only eight participants (1.2%) were diagnosed with hearing loss, and 192 (27.7%) had a family history of hearing loss. Smoking was reported among 65 (9.4%) participants (Table 1).

**Table 1:** Socio-demographic data of study participants, Taif, Saudi Arabia

Socio-demographic data	No	%
Age in years		
< 18	94	13.6%
19-29	410	59.2%
30-39	72	10.4%
40+	117	16.9%
Gender		
Male	147	21.2%
Female	546	78.8%
Nationality		
Saudi	671	96.8%
Non-Saudi	22	3.2%
Have you been diagnosed with hearing loss?		
Yes	8	1.2%
No	685	98.8%
Do you have any relatives affected by hearing loss?		
Yes	192	27.7%
No	501	72.3%
Smoking		
Yes	65	9.4%
No	628	90.6%

ETD symptoms intensity among study participants, Taif, Saudi Arabia. As Table 2 shows the mean score for ear problems when you have a cold or sinusitis was 1.95 ± 1.32 out of 7, followed by feeling that ears are clogged or “underwater (1.93 ± 1.27), ringing in the ears (1.92 ± 1.26), pain in the ears (1.80 ± 1.13), and crackling or popping sounds in the ears (1.78 ± 1.24). The lest score was for feeling pressure in the ears (1.66 ± 1.01). The overall mean score was 1.8 ± 0.9 out of 7. The exact 122 (61%) participants among those who had ETD or hearing loss reported improvement in their symptoms while going to another city below sea level.

**Table 2:** Eustachian tube dysfunction symptoms intensity among study participants, Taif, Saudi Arabia

ETDQ-7	Mean	SD
Pressure in the ears?	1.66	1.01
Pain in the ears?	1.80	1.13
A feeling that your ears are clogged or “under water”?	1.93	1.27
Ear problems when you have a cold or sinusitis?	1.95	1.32
Crackling or popping sounds in the ears?	1.78	1.24
Ringing in the ears?	1.92	1.26
A feeling that your hearing is muffled?	1.77	1.25
Overall mean score	1.8	0.9
If you have been diagnosed with hearing loss or have symptoms of eustachian tube obstruction, have you noticed an improvement in your symptoms while going to another city below sea level?	No	%
Yes	122	61%
No	78	39%

**Figure 1:**
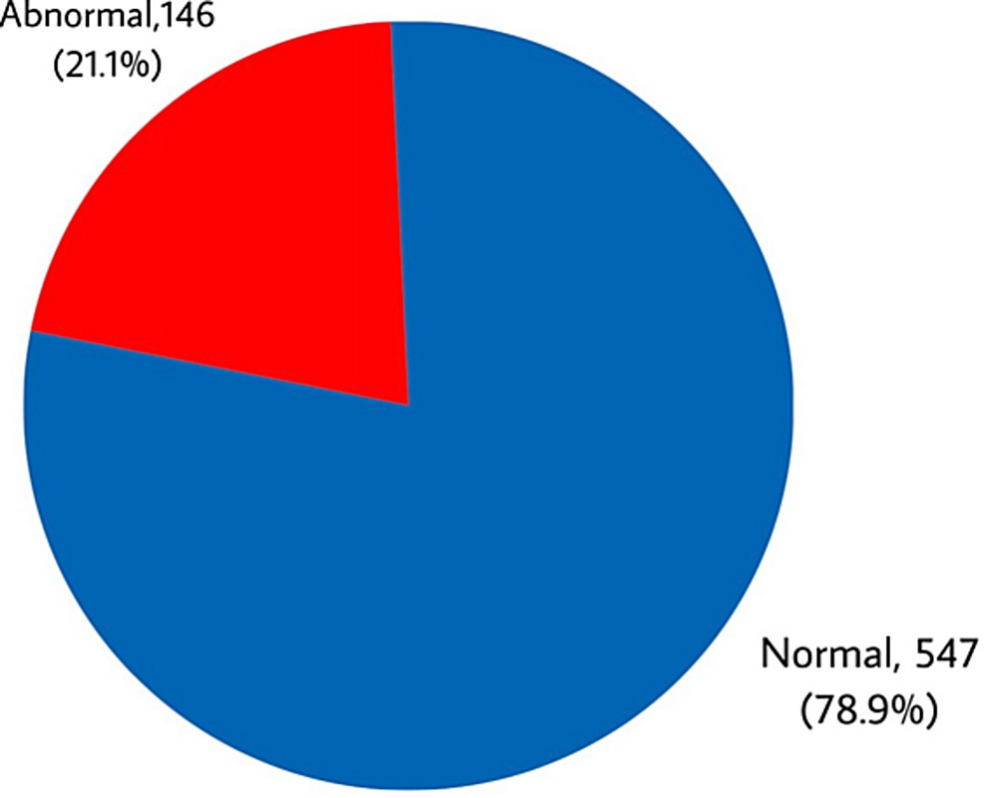
Prevalence of eustachian tube dysfunction among population in Taif city, Saudi Arabia

Prevalence of ETD among the population in Taif city, Saudi Arabia. The exact 146 (21.1%) participants had ETD while 547 (78.9%) had reported normal function (Figure 1).

Distribution of ETD among participants by their socio-demographic data. ETD was insignificantly higher among the young aged group (22.3%) compared to 17.9% of participants aged 40 years or more (P=0.832). On the other hand, 75% of participants diagnosed with hearing loss complained of ETD versus 20.4% of others who did not record statistical significance (P=0.001). Also, 30.2% of participants with a family history of hearing loss had ETD compared to 17.6% of others (P=0.001). ETD was detected among 69.2% of those who were previously diagnosed with ETD versus 20.1% of others (P=0.001) (Table 3).

**Table 3:** Distribution of eustachian tube dysfunction among participants by their socio-demographic data P: Pearson X^2^ test​​​, $: Exact probability test *: P < 0.05 (significant)

Socio-demographic data	Eustachian tube dysfunction				P-value
	Normal		ETD		
	No	%	No	%	
Age in years					.832
< 18	73	77.7%	21	22.3%	
19-29	322	78.5%	88	21.5%	
30-39	56	77.8%	16	22.2%	
40+	96	82.1%	21	17.9%	
Gender					.499
Male	119	81.0%	28	19.0%	
Female	428	78.4%	118	21.6%	
Nationality					.209^$^
Saudi	532	79.3%	139	20.7%	
Non-Saudi	15	68.2%	7	31.8%	
Have you been diagnosed with hearing loss?					.001*^$^
Yes	2	25.0%	6	75.0%	
No	545	79.6%	140	20.4%	
Do you have any relatives affected by hearing loss?					.001*
Yes	134	69.8%	58	30.2%	
No	413	82.4%	88	17.6%	
Have you been diagnosed with Eustachian tube dysfunction?					.001*^$^
Yes	4	30.8%	9	69.2%	
No	543	79.9%	137	20.1%	
Smoking					.169
Yes	47	72.3%	18	27.7%	
No	500	79.6%	128	20.4%	

Logistic regression model for risk factors of ETD among the study population. Among all risk factors, participants who were diagnosed with hearing loss showed 10 times more likely of ETD (OR=10.1; 95% CI: 1.95-53.1), and participants who were diagnosed with ETD showed 9 times likelihood more for ETD (OR=9.47; 95% CI: 2.7-32.9). Also, participants with a family history of hearing loss showed a nearly doubled risk for having ETD (OR=1.98; 95% CI: 1.33-2.94) and smokers had nearly the same doubled likelihood (OR=1.83; 95% CI: 1.01-3.48) (Table 4).

**Table 4:** Logistic regression model for risk factors of Eustachian tube dysfunction among study population ORA: Adjusted odds ratio, CI: confidence interval * P < 0.05 (significant)

Factors	P-value	OR_A_	95% CI	
			Lower	Upper
Age	.126	.84	.68	1.05
Female	.200	1.40	.84	2.35
Non-Saudi	.123	2.09	.82	5.36
Diagnosed with hearing loss	.006*	10.16	1.95	53.01
Family history of hearing loss	.001*	1.98	1.33	2.94
Diagnosed with ETD	.000*	9.47	2.73	32.93
Smoking	.046*	1.83	1.01	3.48

## Discussion

Keeping the middle ear healthy is crucial for normal hearing, which requires maintaining middle ear pressure at near-ambient levels. This is obtained by temporary, muscle-assisted dilations of the Eustachian tube lumen. Though ETD is mostly featured by the lack of sufficiency to adequately open to match pressure between the middle ear and the nasopharynx, it also includes ears with a continuously open, patulous eustachian tube [12,13]. The current study aimed to assess the prevalence and associated factors of ETD in the Taif public.

The study results showed that about one-fifth of the participants (21.1%) had ETD based on ETDQ-7. This is a slightly high prevalence relative to what was reported in the literature but this may be explained by that diagnosis based on a subjective scale depends mainly on participants' perception of the asked questions and their own evaluation but there are many other methods that may be used to confirm the existence of ETD among positive cases with the scale [14-17].

Worldwide, the prevalence of ETD among the adult population is nearly 1% [18], with about 40% of children experiencing at least temporary ETD [19]. Many studies have estimated that among patients undergoing tympanoplasty for middle ear disorders, nearly 70% develop ETD [20,21]. In the USA, Shan et al. [8] estimated that the overall prevalence of ETD among adults in was nearly 4.6%, consistent with a total of 11 million affected individuals. The prevalence was slightly higher among the elderly (5.4%) and among patients with malignancy (9.8%) [5]. A higher prevalence was estimated by Newman et al. [9] who reported that the mean score of ETDQ-7 score for temporomandibular joint disorder patients was 24.5 ± 12.5. Two-thirds of patients had an ETDQ-7 score of >14.5, which indicates clinically significant ETD. Also, Rennie et al. [22] found that ETD was significantly higher in rhinology patients (mean score 3.1 ± 1.64) as compared to a control population (mean 1.3 ± 0.3). In Saudi Arabia, Alshehri et al. [4] estimated higher prevalence among public in Jeddah than reported in the current study which was 42.5%.

Regarding factors associated with ETD among study participants, logistic regression model showed that participants who were diagnosed with hearing loss showed the highest risk for experiencing ETD with 10 folds more risk than other. Also, those who were diagnosed with ETD showed nearly the second highest risk among study population with nearly ninefold more than others who were never diagnosed. Additionally, family history and smoking were associated with doubles risk for developing ETD. Other factors including age and gender were insignificantly associate with ETD among the current study population. These findings were consistent with other literature conclusion about factors associated with developing ETD [4,23].

## Conclusions

Our study revealed the prevalence of ETD and hearing loss among the public in Taif, Saudi Arabia, and the factors associated with ETD. According to our prevalence data, ETD is slightly higher prevalence relative to what was reported in the literature, highlighting the need for additional focus, awareness, and research. The strengths of this study include its large sample size, population-based design, and use of a validated questionnaire to assess ETD. The main limitation of this research includes being a descriptive online questionnaire which may be achieved by certain categories in the community who are educated and have internet access neglecting a wide sector of illiterate and others with no smartphone accessibility which may affect the sample representativeness and generalizability of the results. Also, Inability to ensure that our study covered all residential regions of the city. But the main barrier to doing a direct interview study was the current situation of the COVID-19 pandemic with many social restrictions. Future studies should consider different environmental risk factors when collecting data and attempt to identify an association between hearing loss and ETD.
